# RnRTD: Intelligent Approach Based on the Relationship-Driven Neural Network and Restricted Tensor Decomposition for Multiple Accusation Judgment in Legal Cases

**DOI:** 10.1155/2019/6705405

**Published:** 2019-07-07

**Authors:** Xiaoding Guo, Hongli Zhang, Lin Ye, Shang Li

**Affiliations:** Harbin Institute of Technology, Harbin, China

## Abstract

The use of intelligent judgment technology to assist in judgment is an inevitable trend in the development of judgment in contemporary social legal cases. Using big data and artificial intelligence technology to accurately determine multiple accusations involved in legal cases is an urgent problem to be solved in legal judgment. The key to solving these problems lies in two points, namely, (1) characterization of legal cases and (2) classification and prediction of legal case data. Traditional methods of entity characterization rely on feature extraction, which is often based on vocabulary and syntax information. Thus, traditional entity characterization often requires extensive energy and has poor generality, thus introducing a large amount of computation and limitation to subsequent classification algorithms. This study proposes an intelligent judgment approach called RnRTD, which is based on the relationship-driven recurrent neural network (rdRNN) and restricted tensor decomposition (RTD). We represent legal cases as tensors and propose an innovative RTD method. RTD has low dependence on vocabulary and syntax and extracts the feature structure that is most favorable for improving the accuracy of the subsequent classification algorithm. RTD maps the tensors, which represent legal cases, into a specific feature space and transforms the original tensor into a core tensor and its corresponding factor matrices. This study uses rdRNN to continuously update and optimize the constraints in RTD so that rdRNN can have the best legal case classification effect in the target feature space generated by RTD. Simultaneously, rdRNN sets up a new gate and a similar case list to represent the interaction between legal cases. In comparison with traditional feature extraction methods, our proposed RTD method is less expensive and more universal in the characterization of legal cases. Moreover, rdRNN with an RTD layer has a better effect than the recurrent neural network (RNN) only on the classification and prediction of multiple accusations in legal cases. Experiments show that compared with previous approaches, our method achieves higher accuracy in the classification and prediction of multiple accusations in legal cases, and our algorithm is more interpretable.

## 1. Introduction

In contemporary society, the demand for big data assistance in the judgment of legal cases, such as case intelligence research [1] and judgment [2], big data comprehensive supervision, and assistance in handling legal cases, is increasing with the development of big data and artificial intelligence technology. Researchers are committed to creating an “intelligent legal case judgment” project that combines big data and artificial intelligence. Legal case multiaccusation judgment business is an important part of the realization of such a project. Legal case multiaccusation judgment technology fully applies big data and artificial intelligence technology to service judgment making, legal case handling [3], and facilitation of the public. Big data provides judgments with recognized standards for judging legal cases and avoids the occurrence of different judgment results in similar legal cases. Artificial intelligence technology avoids the subjectivity of human beings, performs scientific and accurate analyses of cases from the perspective of cases and laws, and helps judges make objective judgment in legal cases.

**Figure 1:**
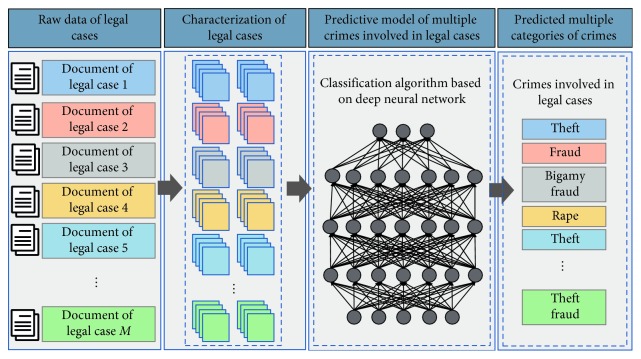
Multiaccusation classification for legal cases.

The solution to using big data and artificial intelligence technology to accurately judge multiple accusations in different legal cases involves two main points, namely, (1) construction of a comprehensive and accurate characterization method of legal cases and (2) realization of a classification and prediction algorithm for multiple accusations involved in a large number of legal cases.  Figure 1 shows the process of multiaccusation classification for legal cases. Traditional methods of entity characterization are often used to model an entity by tagging it. However, these feature extraction methods are highly dependent on the vocabulary and syntax in the entity data set and require heavy manpower and material resources. The generality of the tagged model is poor. In addition, feature extraction methods based on vocabulary and syntax require strong expert knowledge as support. The resulting entity characterization considerably limits the subsequent classification algorithm, and the algorithm's accuracy becomes highly volatile.

**Figure 2:**
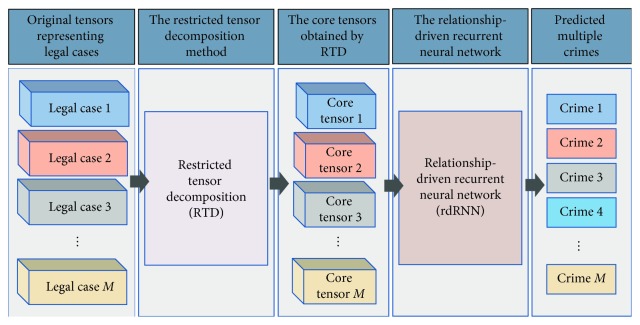
Framework of the proposed RnRTD.

This study proposes an intelligent legal case judgment technique called RnRTD, which is based on the relationship-driven recurrent neural network (rdRNN) and restricted tensor decomposition (RTD).  Figure 2 shows the framework of our approach. We present legal case data as tensor *χ* and propose an RTD technique. RTD is less dependent on vocabulary and syntax than traditional feature extraction methods, and it focuses more on extracting the information of potential structures in legal case tensors. RTD maximizes the accuracy of rdRNN by combining text and structural information. RTD maps legal case tensor *χ* into specified feature space *ℤ*, which decomposes the original tensor *χ* into core tensor $\tilde{\chi }$ and its corresponding feature matrix set {*C*_*k*_} under the restricted condition *η* in RTD. The obtained core tensor $\tilde{\chi }$ represents the tensor structure information that is most helpful in improving the accuracy of the rdRNN classification algorithm. Core tensor $\tilde{\chi }$ can be interpreted as the most advantageous feature structure in *χ* for rdRNN. RTD is an important feature extraction and dimensionality reduction operation. This study uses rdRNN to update and optimize restricted condition *η* in RTD iteratively so that its feature space continually approaches an ideal region, thus enabling rdRNN to achieve an optimal effect in the classification of multiple accusations in legal cases.

Compared with traditional feature extraction methods, RTD obtains legal case characterization containing tensor element and structural information that is more conducive for improving the accuracy of the rdRNN classification algorithm, and it has lower dependence on vocabulary, syntax, and expert knowledge. That is, the RTD legal case characterization model has better universality and fewer requirements on the dataset format in comparison with traditional feature extraction methods. Compared with the direct use of the original legal case tensor *χ* as the input of the RNN classification algorithm, rdRNN with an RTD layer has a better effect on the classification of multiple accusations in legal cases. The main reason is that rdRNN constantly updates and optimizes RTD restricted condition *η*, thereby enabling RTD to point to feature space where rdRNN has the best effect in legal case classification.

The main contributions of this study are summarized in the following points:

- *This study uses a new method of characterizing legal cases.* This study expresses a legal case as a tensor and proposes an RTD method that maps the original legal case tensor into a new feature space. RTD extracts the favorable tensor structure and text information for the subsequent classification algorithm from the original legal case tensor. RTD also extracts valuable tensor features and reduces tensor dimensions. The core tensor obtained by RTD is interpreted as the most valuable tensor structure and textual feature information extracted from the original legal case tensor for the rdRNN classification algorithm.
- *This study proposes rdRNN, which is a new approach for intelligent judgment of multiple accusations in legal cases.* We add a new gate and a similar case list to control the interaction between tensors of legal cases on the basis of the original neural networks. rdRNN is particularly used for the intelligent judgment of multiple accusations in legal cases. It fully considers the impact of the relationship between legal cases on the judgment results of such cases. For example, highly similar legal cases are likely to have similar judgment results and vice versa.
- *This study proposes a neural network-based method for the optimization of the restricted tensor.* The restricted tensor is a bridge between the RTD algorithm and rdRNN. rdRNN controls the tensor decomposition process by optimizing the restricted tensor, which guides the core tensor along the direction that is most conducive for improving the accuracy of the classification model. We derive the partial derivative of the loss function in rdRNN for the restricted tensor and realize the optimization operation of the neural network for the restricted tensor.

Section 2 gives the recent research progress on the classification of multiple crimes in legal cases.  Section 3 introduces related definitions and the concepts involved in this study.  Section 4 introduces the proposed approach for the judgment of multiple accusations in legal cases.  Section 5 provides the experimental results and analysis of this study, and Section 6 presents a detailed discussion of the proposed method.

## 2. Related Work

With the advent of the era of big data and the development of artificial intelligence technology [4], the emergence of deep neural networks provides great prospects for accurate classification and prediction [5]. Neural network-based knowledge representation and reasoning methods enable deep learning approaches to be applied to many scenarios [6]. For the legal field, the combination of artificial intelligence and law has become an inevitable trend [7]. However, current research in this area mainly focuses on legal case modeling [8], legal case document retrieval [9], legal consultation question-and-answer systems [10], and legal case similarity reasoning work [2]. Little research has been conducted on the multiaccusation determination of cases in the legal field.

Bartolini et al. proposed a semantic annotation method for indexing and retrieving legal texts [11]. The method uses a specific segment extraction and text classification algorithm to automatically semantically mark legal documents. Aleven developed a computational model based on artificial intelligence algorithms and professional legal knowledge [2]. The model determines the correlation between cases based on the context and problem scenarios of the case. Joshi et al. proposed a text mining method for electronic evidence review of legal cases [12]. The method uses semantic topic and text classification technology to repeatedly detect the feature vocabulary in legal documents and then automatically segments and screens the documents, avoiding the manual work of legal analysts.

Sulea et al. proposed a legal case judgment system based on SVM classifier [13]. The method uses machine learning techniques to predict the legal field to which the legal case belongs and the outcome of its judgment. By accurately extracting the features of legal cases, the method can roughly predict the specific date of the case. Brninghaus and Ashley proposed a text classification method based on facts of legal cases [14]. The method uses artificial intelligence algorithms and legal background knowledge to predict the outcome of legal cases. The method extracts facts of legal cases, indexes and models them according to the features, and finally completes the classification of legal cases.

The critical part for the prediction of legal case judgments is case modeling and case classification. Traditional text modeling methods are based on feature tags, which rely heavily on the syntax and semantic information of the source data. Labeling features requires a lot of manual work and expert knowledge. Therefore, the text classification algorithm formed on this basis is not scalable, and the accuracy is highly volatile.

## 3. Preliminaries

This section introduces the related methods, definitions, and background knowledge involved in this study.  Section 3.1 presents the basic notations and definitions.  Section 3.2 provides a formal representation of the tensor decomposition problem.  Section 3.3 introduces the calculation process of forward propagation in bidirectional long short-term memory (Bi-LSTM).  Section 3.4 presents a formal description of the problem about intelligent legal judgment to be solved in this study.

### 3.1. Definitions and Notations

This section describes the relevant notations and definitions required in this work. Tensors are actually multidimensional matrices [15], which we represent in Euler script letters, such as *χ* and *ν*. We refer to the dimensions as tensor modes and to the number of a tensors modes as order. We describe the scalars in lowercase letters (such as *a*, *b*) and the vectors in boldface lowercase letters (such as *c*, *d*). We declare the matrices in capital letters, such as *A* and *B*. We use *A*^T^ to represent the transpose of matrix *A*. We express the identity matrix as *I*, the identity tensor as *τ*, and the matrix with all elements of 1 as 1.  Table 1 shows all the required notations and definitions.

**Definition 1 (outer product).**

The outer product of vectors **a** ∈ ^*I*^ and **b** ∈ ^*J*^ is denoted as *A*=**a**∘**b**, where *A* ∈ *ℝ*^*I*×*J*^ and *A*(*i*, *j*)=**a**(*i*)**b**(*j*).

**Definition 2 (elementwise multiplication).**

The elementwise multiplication of vectors **a** ∈ ^*I*^ and **b** ∈ ^*I*^ is denoted as *A*=**a***∗ ***b**, where *A* ∈ ^*I*^ and *A*(*i*)=**a**(*i*)**b**(*i*). In another case, the elementwise multiplication of vector **a** ∈ ^*I*^ and matrix *A* ∈ ^*I*×*J*^ is denoted as *Z*=**a***∗A*, where *Z* ∈ ^*I*×*J*^ and *Z*(*i*, *j*)=**a**(*i*)*A*(*i*, *j*).

**Definition 3 (Kronecker product).**

Given vectors **a** ∈ ^*I*^ and **b** ∈ ^*J*^, their Kronecker product is denoted as *S*=**a** ⊗ **b**, where *S* ∈ ^*IJ*^ and *S*=[**a**(1)**b**^T^, **a**(2)**b**^T^, ⋯,**a**(*I*)**b**^T^]^T^. Given matrices *A* ∈ ^*I*×*J*^ and *B* ∈ ^*P*×*Q*^, their Kronecker product is denoted as *A* ⊗ *B*.

(1) $$\begin{array}{c} A⊗B=\left[A\left(:,1\right)⊗B\left(:,1\right)A\left(:,1\right)⊗B\left(:,2\right)\cdots A\left(:,J\right)⊗B\left(:,Q-1\right)A\left(:,J\right)⊗B\left(:,Q\right)\right]. \end{array}$$

**Definition 4 (Khatri–Rao product).**

Given matrices *A* ∈ ^*I*×*R*^ and *B* ∈ ^*J*×*R*^, their Khatri–Rao product is denoted as *A*⊙*B*, which is calculated by combining the Kronecker product of each corresponding column in *A* and *B*, that is

(2) $$\begin{array}{c} A⊙B=\left[A\left(:,1\right)⊗B\left(:,1\right)\cdots A\left(:,r\right)⊗B\left(:,r\right)\cdots A\left(:,R\right)⊗B\left(:,R\right)\right]. \end{array}$$

**Definition 5 (*n*-mode matricization).**

Given an *N*-mode tensor *χ*, *χ* ∈ *ℝ*^*I*_1_×*I*_2_×⋯×*I*_*N*_^. *χ* can be matrixed into *N* forms according to each mode. We denote the n-mode matricization of *χ* as *χ*_(*n*)_, where *χ*_(*n*)_ ∈ ^*I*_*n*_×*I*_1_⋯*I*_*n*−1_*I*_*n*+1_⋯*I*_*N*_^. *χ*_(*n*)_ is obtained by keeping the nth mode unchanged while expanding and concatenating the slices of the remaining modes into a matrix.

**Definition 6 (Frobenius norm of a tensor).**

Given an N-mode tensor *χ*, *χ* ∈ *ℝ*^*I*_1_×*I*_2_×⋯×*I*_*N*_^, the Frobenius norm of *χ* is denoted as

(3) $$\begin{array}{c} {\left|\left|\chi \right|\right|}_{\text{F}}=\sqrt{\overset{I_{1}}{\underset{i_{1}}{\sum }}\overset{I_{2}}{\underset{i_{2}}{\sum }}\cdots \overset{I_{N}}{\underset{i_{N}}{\sum }}\chi {\left(i_{1},i_{2},\ldots ,i_{N}\right)}^{2}}. \end{array}$$

**Definition 7 (*n*-mode stretch).**

Given an *N*-mode tensor *ν*, *ν* ∈ *ℝ*^*J*_1_×*J*_2_×⋯×*J*_*N*_^, and a weight matrix *W*, *W* ∈ *ℝ*^*J*_*n*_×∏_*k*≠*n*_^*N*^*J*_*k*_^. The *n*-mode stretch between *ν* and *W* is expressed as *υ*×^*n*^*W*=*κ*, where *κ* ∈ *ℝ*^*J*_1_×*J*_2_×⋯×*J*_*N*_^.

(4) $$\begin{array}{c} \kappa \left(j_{1},j_{2},\ldots ,j_{n},\ldots ,j_{N}\right)=W\left(j_{1},\overset{N}{\underset{k\neq n}{\prod }}j_{k}\right)\upsilon \left(j_{1},j_{2},\ldots ,j_{n},\ldots ,j_{N}\right). \end{array}$$

**Definition 8 (*n*-mode product).**

Given an *N*-mode tensor *χ* ∈ *ℝ*^*I*_1_×*I*_2_×⋯×*I*_*N*_^ and a matrix *C* ∈ ^*I*_*n*_×*J*^, their *n*-mode product is denoted as *λ*=*χ*×_*n*_*C*, *λ* ∈ *ℝ*^*I*_1_×⋯×*I*_*n*−1_×*J*×*I*_*n*+1_×⋯×*I*_*N*_^.

(5) $$\begin{array}{c} \lambda \left(i_{1},\ldots ,i_{n-1},j,i_{n+1},\ldots ,i_{N}\right)=\overset{I_{n}}{\underset{m=1}{\sum }}\chi \left(i_{1},\ldots ,i_{n-1},m,i_{n+1},\ldots ,i_{N}\right)C\left(m,j\right). \end{array}$$

### 3.2. Tensor Decomposition

**Figure 3:**
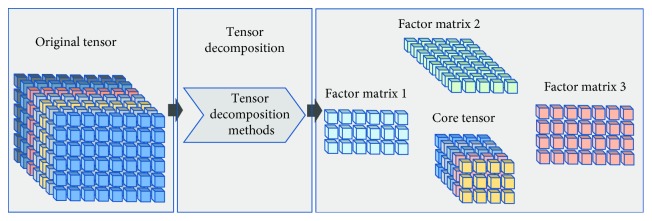
Tensor decomposition.

Many tensor decomposition methods, such as PARAFAC and Tucker decomposition, are currently available [15]. As shown in Figure 3, tensor decomposition methods decompose the original tensor into a core tensor and a series of corresponding factor matrices. The essence of tensor decomposition is to approximate the original tensor by using the product of the core tensor and the factor matrices. The mathematical description of tensor decomposition is as follows:

Given an *N*-mode tensor *χ*, *χ* ∈ *ℝ*^*I*_1_×*I*_2_×⋯×*I*_*N*_^. The following formula can be obtained by using the tensor decomposition method:

(6) $$\begin{array}{c} \chi \approx \tau \times_{1}C_{1}\times_{2}C_{2}\times \cdots \times_{N}C_{N}, \end{array}$$

where *τ* is the core tensor, *τ* ∈ *ℝ*^*J*_1_×*J*_2_×⋯×*J*_*N*_^, and {*C*_*n*_} is the corresponding factor matrix set, *C*_*n*_ ∈ *ℝ*^*J*_*n*_×*I*_*n*_^. Each element in {*C*_*n*_} is a column orthogonal matrix. *τ* and {*C*_*n*_} also minimize function *φ*, where

(7) $$\begin{array}{c} \varphi ={\left\|\chi -\tau \overset{N}{\underset{n}{\prod }}\times_{n}C_{n}\right\|}_{\text{F}}. \end{array}$$

### 3.3. Bi-LSTM

RNNs have far-reaching implications for the study of sequence data [16]. The nodes between the hidden layers of RNN are connected [17], that is, the input of the hidden layer contains not only the output of the input layer but also the output of the hidden layer at the last moment. In theory, RNN can process sequence data of any length. However, gradient disappearance or gradient explosion often occurs when RNN deals with long-distance dependence, thereby making RNN training difficult. The hidden layers of the original RNN has only one kind of state, which is very sensitive to short-term inputs. Long short-term memory (LSTM) deals with long-distance dependence by increasing the long-term memory state in the original RNN [18].

**Figure 4:**
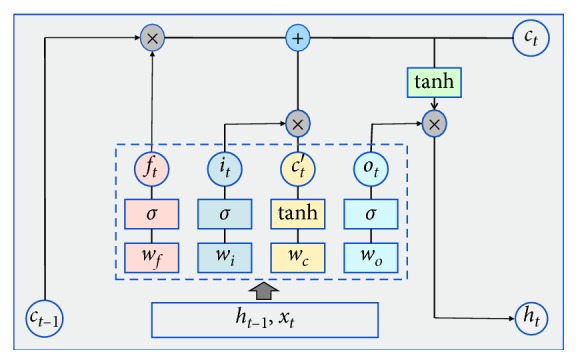
Framework of LSTM.

As shown in Figure 4, we represent the input value of LSTM at time *t* as *x*_*t*_, the output value from the previous moment *t* − 1 as *h*_*t*−1_, and the long-term unit state at time *t* − 1 as *c*_*t*−1_. We record the unit status entered at time *t* as ${\tilde{c}}_{t}$. The output value of LSTM at time *t* comprises two parts, namely, the output value of LSTM at current time *h*_*t*_ and the unit state of current time *c*_*t*_. LSTM sets up three control gates, which are forget, input, and output, to control the long-term unit state *c*. The forget gate is used to determine how much of the long-term unit state at the previous moment is retained at the current moment. For example, the forget gate *f*_*t*_ at time *t* determines the weight of *c*_*t*−1_ in the calculation of *c*_*t*_. The input gate is used to determine how much of the input of LSTM is retained in the current long-term unit state. For example, input gate *i*_*t*_ determines the weight that *x*_*t*_ takes while calculating *c*_*t*_. The output gate is used to determine how much the long-term unit state at the current moment contributes to the output of LSTM at the current time. For example, output gate *o*_*t*_ determines the influence of the value of *c*_*t*_ on *h*_*t*_.

The process of forward propagation calculation in LSTM is described as follows:

(8) $$\begin{array}{c} f_{t}=\sigma \left(w_{f}\cdot {\left[h_{t-1},x_{t}\right]}^{\text{T}}+b_{f}\right),\\ i_{t}=\sigma \left(w_{i}\cdot {\left[h_{t-1},x_{t}\right]}^{\text{T}}+b_{i}\right),\\ {\tilde{c}}_{t}=\tanh\left(w_{c}\cdot {\left[h_{t-1},x_{t}\right]}^{\text{T}}+b_{c}\right),\\ o_{t}=\sigma \left(w_{o}\cdot {\left[h_{t-1},x_{t}\right]}^{\text{T}}+b_{o}\right). \end{array}$$

The long-term unit state *c*_*t*_ at current time *t* is calculated by *f*_*t*_, *i*_*t*_, *c*_*t*−1_ and ${\tilde{c}}_{t}$, and the final output of LSTM *h*_*t*_ is calculated by *o*_*t*_ and *c*_*t*_. That is,

(9) $$\begin{array}{c} c_{t}=f_{t}*c_{t-1}+i_{t}*{\tilde{c}}_{t},\\ h_{t}=o_{t}*\;\;\tanh\left(c_{t}\right), \end{array}$$

where *h*_*t*−1_ is the output of LSTM at time *t* − 1, *x*_*t*_ is the input of LSTM at time *t*, *σ* is the sigmoid function, which is our selected activation function in LSTM, ${\tilde{c}}_{t}$ is the unit state input at time *t*, *w*_*f*_, *w*_*i*_, and *w*_*o*_ are the weight matrices of the forget gate *f*_*t*_, the input gate *i*_*t*_, and the output gate *o*_*t*_, respectively, and *b*_*f*_, *b*_*i*_, and *b*_*o*_ are the bias matrices of *f*_*t*_, *i*_*t*_, and *o*_*t*_, respectively. The activation function used in calculating ${\tilde{c}}_{t}$ is the hyperbolic tangent function, where *w*_*c*_ is the weight matrix and *b*_*c*_ is the bias term.

Bi-LSTM is a bidirectional RNN [19]. The unit state of the hidden layer in Bi-LSTM is calculated from the outputs of forward and backward LSTM. We define the output unit state of Bi-LSTM at time *t* as *h*_Bi − LSTM_*t*__, the output unit state of forward LSTM as *h*_*f*_*t*__, and the output unit state of backward LSTM as *h*_*b*_*t*__. The aforementioned forward propagation formula of LSTM implies that

(10) $$\begin{array}{c} h_{f_{t}}={\text{LSTM}}^{\to }\left(h_{f_{t-1}},x_{t}\right),\\ h_{b_{t}}={\text{LSTM}}^{\leftarrow }\left(h_{b_{t+1}},x_{t}\right),\\ h_{{\text{Bi}-\text{LSTM}}_{t}}=\left[h_{f_{t}},h_{b_{t}}\right]. \end{array}$$

### 3.4. Problem Description

**Problem 1 .**

We express the legal case as a tensor and classify the legal case according to the judgment result. The category of each legal case is indicated by a scalar, such as *r*. Given a legal case dataset *Ω* that contains legal cases with judgment results, *Ω*={(*χ*^(1)^, *r*^(1)^), (*χ*^(2)^, *r*^(2)^), ⋯, (*χ*^(*N*)^, *r*^(*N*)^)}. *χ*^(*n*)^ represents the nth legal case in the legal case dataset *Ω*. *r*^(*n*)^ indicates the type of legal judgment result that corresponds to the nth legal case. Our goal is to train a case classification model *ϕ*(*χ*) that can classify legal cases based on their judgment results.

**Figure 5:**
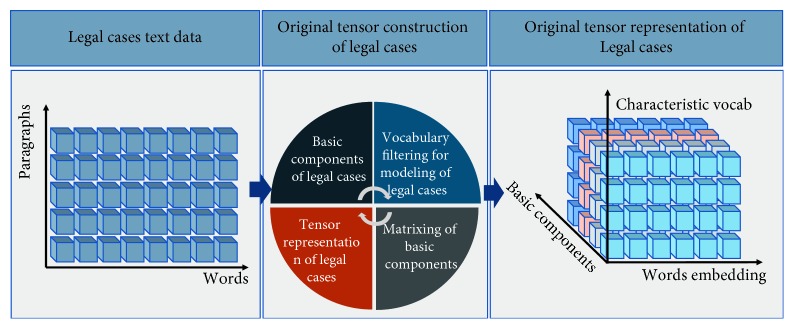
Original tensor that represents the legal case.

In this study, legal cases are represented as three-dimensional tensors. As shown in Figure 5, the first dimension represents the basic components of the case, such as the defendant's statement, the plaintiff's statement, the public prosecution, and the court's trial. On this basis, the matrix slice that contains the last two dimensions represents the matrix form of the corresponding legal case component. The matrix slice is composed of the accumulation of word vectors inside the legal case component. Generally, case components are matrixed instead of including the word vectors of all the words in the matrix. We selectively extract words that are valuable for the legal case classification. These words can be divided into two categories. The first category usually includes nouns or pronouns, such as characters, times, places, and objects; the second category usually comprises adjectives, numerals, or verbs, such as the means of committing accusations, the degree of harm, and the number of accusations.

## 4. Our Approach

**Figure 6:**
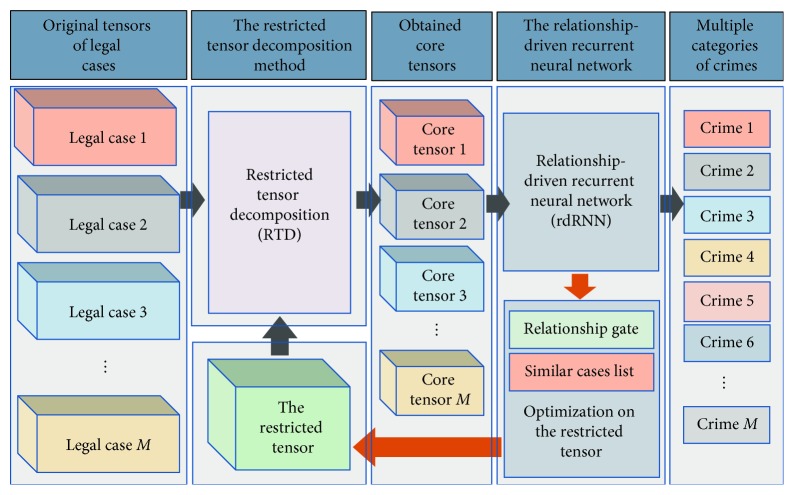
Framework of the RnRTD method.

This study proposes RnRTD for the multiaccusation determination of legal cases.  Figure 6 shows the RnRTD framework. First, we extract core tensors from the original tensors using the RTD method. The core tensor approximates the restricted tensor in terms of the tensor structure and elements. Second, we use rdRNN to optimize the restricted tensor so that it guides the core tensor along the direction that is most conducive for improving the accuracy of the classification model.

### 4.1. RTD Method

This study proposes a new tensor decomposition method called RTD method. The inputs of the RTD algorithm include the restricted condition tensor *η* and tensor *χ* that represents the legal case. The RTD outputs include core tensor $\tilde{\chi }$ and its corresponding factor matrix sets, namely, {*C*_*k*_} and {*D*_*k*_}. RTD decomposes *χ* into a core tensor $\tilde{\chi }$ under the action of the restricted condition *η*. $\tilde{\chi }$ is approximated to *η* in terms of tensor structure and internal element values. RTD can be interpreted as a mapping of the original tensor *χ* to the core tensor $\tilde{\chi }$. In short, RTD achieves directional decomposition of tensors and extracts vital information from tensors while reducing their dimension. In this study, we define core tensor $\tilde{\chi }$ as the most favorable tensor structure and element value information for the subsequent legal case classification algorithm, namely, rdRNN. On this basis, we construct a deep neural network model for RnRTD that is dedicated to legal intelligence judgments.

**Figure 7:**
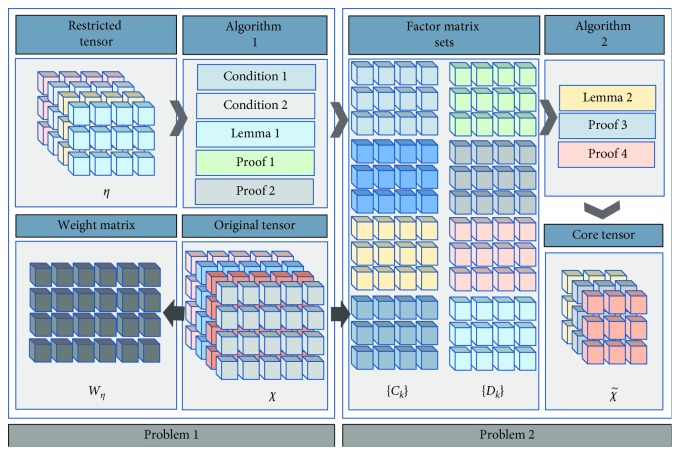
Framework of the RTD method.

RTD decomposes the original tensor under the restricted condition so that the obtained core tensor constantly approaches the restricted tensor in terms of tensor structure and element value. In Figure 7, the formal description of the problems to be solved by the RTD algorithm is shown as Problems 1 and 2.

**Problem 2 .**

Given tensor *χ* ∈ *ℝ*^*I*_1_×*I*_2_×⋯×*I*_*K*_^, restricted tensor *η* ∈ *ℝ*^*J*_1_×*J*_2_×⋯×*J*_*K*_^, and its weight *w*_*η*_, we derive two factor matrix sets, namely, {*C*_*k*_} and {*D*_*k*_}, *C*_*k*_ ∈ ^*I*_*k*_×*H*_*k*_^, *D*_*k*_ ∈ ^*J*_*k*_×*H*_*k*_^, that {*C*_*k*_} and {*D*_*k*_} minimize the following function:

(11) $$\begin{array}{c} \phi ={\left\|\chi \overset{K}{\underset{k}{\prod }}\times_{k}C_{k}-W_{\eta }\times^{n}\eta \overset{K}{\underset{k}{\prod }}\times_{k}D_{k}\right\|}_{\text{F}}^{2}. \end{array}$$

Matrix *W*_*η*_ is preset according to the legal case, *W*_*η*_ ∈ *ℝ*^*H*_*n*_×∏_*k*≠*n*_^*K*^*H*_*k*_^. The elements in sets {*C*_*k*_} and {*D*_*k*_} are orthogonal matrices, that is, they meet the following conditions. For any elements *C*_*k*_ and *D*_*k*_ in sets {*C*_*k*_} and {*D*_*k*_},

(12) $$\begin{array}{c} \left\{\begin{array}{c} C_{k}C_{k}^{\text{T}}=I,\\ C_{k}^{\text{T}}C_{k}=I, \end{array}\right.\\ \left\{\begin{array}{c} D_{k}D_{k}^{\text{T}}=I,\\ D_{k}^{\text{T}}D_{k}=I. \end{array}\right. \end{array}$$

In this study, we use the alternating least squares (ALS) algorithm to determine the solution of the objective function *ϕ*. The ALS algorithm can be divided into four steps: (1) randomly pick a variable as a parameter and randomly generate the values of other variables, (2) determine the partial derivative of the loss function *ϕ* in the specified parameter while fixing the values of other variables, (3) set the partial derivative of *ϕ* to the specified parameter as zero and calculate the value of the specified parameter, and (4) select another variable as a parameter and return to Step (2). The ALS algorithm continues to iterate Steps (2), (3), and (4) until the error of the loss function *ϕ* reaches the tolerable upper limit.

Problem 2 needs to be solved using Lemma 1. The specific definition and proof of Lemma 1 are provided as follows.

**Lemma 1 .**

Given function tr(*α*^T^*α*)=∑_*i*_1__^*I*_1_^∑_*i*_2__^*I*_2_^⋯∑_*i*_*n*__^*I*_*N*_^*α*(*i*_1_, *i*_2_,…,*i*_*n*_)^2^, *α* ∈ *ℝ*^*I*_1_×*I*_2_×⋯×*I*_*N*_^, *α* can be a vector, matrix, and tensor. The target function *φ*=tr((*χ*∏_*k*_^*K*^×_*k*_*C*_*k*_)^T^(*χ*∏_*k*_^*K*^×_*k*_*C*_*k*_)), where *χ* ∈ *ℝ*^*I*_1_×*I*_2_×⋯×*I*_*K*_^, *C*_*k*_ ∈ *ℝ*^*I*_*k*_×*H*_*k*_^, and *C*_*k*_ satisfy equation (12). For any element *C*_*k*_0__ in {*C*_*k*_}, *k*_0_ ∈ [1, *K*], the partial differential of function *φ* to *C*_*k*_0__ is ∂*φ*/∂*C*_*k*_0__=*ε*(*χ*∏_*k*≠*k*_0__^*K*^×_*k*_*C*_*k*_)^T^(*χ*∏_*k*_^*K*^×_*k*_*C*_*k*_), where ε is a constant.

The proof of Lemma 1 is shown in Proof 1.

**Proof**

We use *μ* to represent *χ*∏_*k*≠*k*_0__^*K*^×_*k*_*C*__*k*__; it can can be derived that *φ*=tr((*μ*×_*k*_0__*C*_*k*_0__)^T^(*μ*×_*k*_0__*C*_*k*_0__)). We abbreviate the target function *φ*=tr((*μC*_*k*_0__)^T^(*μC*_*k*_0__))=tr(*C*_*k*_0__^T^*μ*^T^*μC*_*k*_0__). We use *ν* to represent *μ*^T^*μ*, and we can get that *φ*=tr(*C*_*k*_0__^T^*νC*_*k*_0__). According to the function derivation rule, we can obtain the following equation: ∂*φ*/∂*C*_*k*_0__=∂tr(*C*_*k*_0__^T^*νC*_*k*_0__)/∂*C*_*k*_0__=*νC*_*k*_0__+*ν*^T^*C*_*k*_0__. Since *ν*=*ν*^T^, the calculation formula for the partial derivative of the function *φ* to *C*_*k*_0__ is

(13) $$\begin{array}{c} \frac{\partial \varphi }{\partial C_{k_{0}}}=2\nu C_{k_{0}}=\varepsilon {\left(\chi \overset{K}{\underset{k\neq k_{0}}{\prod }}\times_{k}C_{k}\right)}^{\text{T}}\left(\chi \overset{K}{\underset{k}{\prod }}\times_{k}C_{k}\right), \end{array}$$

where *ε* is a constant, *ε*=2.

According to the iterative process of the ALS algorithm, the precondition for solving the value of {*C*_*k*_} and {*D*_*k*_} which minimize the function *ϕ* in equation (11) using the ALS algorithm is to calculate the value of ∂*ϕ*/∂*C*_*k*_0__, where *k*_0_ ∈ [1, *K*]. Equation (11) shows that ∂*ϕ*/∂*C*_*k*_0__ and ∂*ϕ*/∂*D*_*k*_0__ are solved in the same manner. Proof 2 provides mathematical proof of the calculation of ∂*ϕ*/∂*C*_*k*_0__.

**Proof**

We use *λ* and *ϖ* to represent *χ*∏_*k*≠*k*_0__^*K*^×_*k*_*C*_*k*_ and *w*_*η*_*η*∏_*k*_^*K*^×_*k*_*D*_*k*_, respectively. According to formula (11), we can obtain that *ϕ*=tr((*λ*×_*k*_0__*C*_*k*_0__ − *ϖ*)^T^(*λ*×_*k*_0__*C*_*k*_0__ − *ϖ*)). We abbreviate the aforementioned formula as *ϕ*=tr((*λC*_*k*_0__ − *ϖ*)^T^(*λC*_*k*_0__ − *ϖ*)). Then, we can determine the following formula: ∂*ϕ*/∂*C*_*k*_0__=(∂tr(*ϖ*^T^*ϖ*) − 2∂tr(*C*_*k*_0__^T^*λ*^T^*ϖ*)+∂tr(*C*_*k*_0__^T^*λ*^T^*λC*_*k*_0__))/∂*C*_*k*_0__. According to the function derivation rule, we derive that ∂tr(*ϖ*^T^*ϖ*)/∂*C*_*k*_0__=0, (∂tr(*C*_*k*_0__^T^*λ*^T^*ϖ*)/∂*C*_*k*_0__)=*λ*^T^*ϖ*. In combination with Lemma 1, we can obtain that (∂tr(*C*_*k*_0__^T^*λ*^T^*λC*_*k*_0__)/∂*C*_*k*_0__)=2*λ*^T^*λC*_*k*_0__. Finally, the following formula is determined:

(14) $$\begin{array}{c} \frac{\partial \phi }{\partial C_{k_{0}}}=-2\lambda^{\text{T}}\varpi +2\lambda^{\text{T}}\lambda C_{k_{0}}. \end{array}$$

We set the value of equation (14) to 0 and obtain that

(15) $$\begin{array}{c} \lambda C_{k_{0}}=\varpi . \end{array}$$

Let *Z*=*λ*_(*k*_0_)_^T^*ϖ*_(*k*_0_)_, then *C*_*k*_0__^T^*Z*=(*λC*_*k*_0__)_(*k*_0_)_^T^*ϖ*_(*k*_0_)_=*ϖ*_(*k*_0_)_^T^*ϖ*_(*k*_0_)_. By combining Equation (12), we derive that

(16) $$\begin{array}{c} \left\{\begin{array}{c} C_{k_{0}}^{\text{T}}Z=Z^{\text{T}}C_{k_{0}},\\ Z=C_{k_{0}}Z^{\text{T}}C_{k_{0}}. \end{array}\right. \end{array}$$

We use the SVD matrix decomposition method to decompose *Z*, and find that *Z*=*PSQ*^T^. *P* and *Q* are orthogonal matrices, *P* is a left singular matrix, *Q* is a right singular matrix, and *S* is a diagonal matrix. After this analysis, the following solution can be obtained:

(17) $$\begin{array}{c} C_{k_{0}}=PQ^{\text{T}}. \end{array}$$

In summary, according to equations (11)–(17), we can derive a solution of ∂*ϕ*/∂*C*_*k*_0__ and *C*_*k*_0__ which are described in equations (14) and (17), respectively. ∂*ϕ*/∂*D*_*k*_ is calculated in the same manner as ∂*ϕ*/∂*C*_*k*_. On this basis, we calculate the value of {*C*_*k*_} and {*D*_*k*_} , which minimize the objective function *ϕ* in formula (11), by using the ALS algorithm.

Algorithm 1 shows the solution of Problem 2 by using ALS algorithm. The inputs of Algorithm 1 are *χ* which represents one legal case, the restricted tensor *η*, and its weight *w*_*η*_. In line 2, we randomly initialize the values of {*C*_*k*_}, {*U*_*k*_}. max_iterations in line 2 represents the maximum number of iterations of ALS algorithm. The function calcu_*Z* in line 4 corresponds to equation (16). Line 5 and 6 show the calculation process of equation (17).

Another problem to be solved by the RTD algorithm is Problem 3, which is the formal description of the process of tensor decomposition on the original tensor under the action of the restricted tensor and its weight. On the basis of Problem 2, we can obtain factor matrix sets {*C*_*k*_} and {*D*_*k*_}, which minimize the value of function *ϕ* in formula (11) while satisfying formula (12).

**Problem 3 .**

Given a tensor *χ* ∈ *ℝ*^*I*_1_×*I*_2_×⋯×*I*_*K*_^ and factor matrix sets {*C*_*k*_} and {*D*_*k*_}, *C*_*k*_ ∈ ^*I*_*k*_×*H*_*k*_^, *D*_*k*_ ∈ ^*J*_*k*_×*H*_*k*_^, {*C*_*k*_} and {*D*_*k*_} are derived from Problem 2. A core tensor $\tilde{\chi }$ is determined, where $\tilde{\chi }\in {\mathbb{R}}^{J_{1}\times J_{2}\times \cdots \times J_{K}}$ and $\tilde{\chi }$ minimize the following target function:

(18) $$\begin{array}{c} F_{\text{RTD}}={\left\|\chi \overset{K}{\underset{k}{\prod }}\times_{k}C_{k}-\tilde{\chi }\overset{K}{\underset{k}{\prod }}\times_{k}D_{k}\right\|}_{\text{F}}^{2}. \end{array}$$

Problem 3 needs to be solved using Lemma 2. The specific definition and proof process of Lemma 2 are as follows.

**Lemma 2 .**

Given the target function $\psi =\text{tr}\left(\left(\tilde{\chi }\prod_{k}^{K}\times_{k}D_{k}\right)\prod_{k}^{K}\times_{k}D_{k}^{\text{T}}{\tilde{\chi }}^{\text{T}}\right)$, $\tilde{\chi }\in {\mathbb{R}}^{J_{1}\times J_{2}\times \cdots \times J_{K}}$, *D*_*k*_ ∈ ^*J*_*k*_×*H*_*k*_^, each element in {*D*_*k*_} satisfies formula (12). Then, the partial derivative of the target function *ψ* to *D*_*k*_0__ where *k*_0_ ∈ [1, *K*] is ∂*ψ*/∂*D*_*k*_0__, $\partial \psi /\partial D_{k_{0}}=\varepsilon \left(\tilde{\chi }\prod_{k}^{K}\times_{k}D_{k}\right)\prod_{k}^{K}\times_{k}D_{k}^{\text{T}}$, where *ε* is a constant.

**Proof**

We use *κ* to represent *τ*∏_*k*_^*K*^×_*k*_*D*_*k*_; *τ* is the identity tensor, *τ* ∈ *ℝ*^*J*_1_×*J*_2_×⋯*J*_*K*_^. Then, the target function *ψ* can be rewritten as $\psi =\text{tr}\left(\tilde{\chi }\kappa \kappa^{\text{T}}{\tilde{\chi }}^{\text{T}}\right)$. We use ρ to represent *κκ*^T^. We can obtain that $\partial \phi /\partial \tilde{\chi }=\partial \text{tr}\left(\tilde{\chi }\kappa \kappa^{\text{T}}{\tilde{\chi }}^{\text{T}}\right)/\partial \tilde{\chi }=\partial \text{tr}\left(\tilde{\chi }\rho {\tilde{\chi }}^{\text{T}}\right)/\partial \tilde{\chi }$. From the function derivation rule, we can get the following formula: $\partial \text{tr}\left(\tilde{\chi }\kappa \kappa^{\text{T}}{\tilde{\chi }}^{\text{T}}\right)/\partial \tilde{\chi }=\partial \text{tr}\left(\tilde{\chi }\rho {\tilde{\chi }}^{\text{T}}\right)/\partial \tilde{\chi }={\left(\rho {\tilde{\chi }}^{\text{T}}+\rho^{\text{T}}{\tilde{\chi }}^{\text{T}}\right)}^{\text{T}}$; thus,

(19) $$\begin{array}{c} \frac{\partial \phi }{\partial \tilde{\chi }}=2\tilde{\chi }\kappa \kappa^{\text{T}}=\varepsilon \left(\tilde{\chi }\overset{K}{\underset{k}{\prod }}\times_{k}D_{k}\right)\overset{K}{\underset{k}{\prod }}\times_{k}D_{k}^{\text{T}}, \end{array}$$

where *ε* is a constant, *ε*=2.

After the aforementioned analysis, Proof 4 gives the solution to Problem 3 and its mathematical proof process while combining Lemma 2 and Proof 3.

**Proof**

We use *υ* to represent *χ*∏_*k*_^*K*^×_*k*_*C*_*k*_ and *γ* to represent *τ*∏_*k*_^*K*^×_*k*_*D*_*k*_, where *τ* is the identity tensor, *τ* ∈ *ℝ*^*J*_1_×*J*_2_×⋯*J*_*K*_^. Then, the function *F*_RTD_ can be rewritten as $F_{\text{RTD}}={\left\|\upsilon -\tilde{\chi }\gamma \right\|}_{\text{F}}^{2}$, that is $F_{\text{RTD}}=\text{tr}\left({\left(\upsilon -\tilde{\chi }\gamma \right)}^{\text{T}}\left(\upsilon -\tilde{\chi }\gamma \right)\right)$. Known by the definition of function tr, $\text{tr}\left(\gamma^{\text{T}}{\tilde{\chi }}^{\text{T}}\upsilon \right)=\text{tr}\left({\tilde{\chi }}^{\text{T}}\upsilon \gamma^{\text{T}}\right)$. Then, we can get the following equation: $\partial F_{\text{RTD}}/\partial \tilde{\chi }=\left(\partial \text{tr}\left(\upsilon^{\text{T}}\upsilon \right)-2\partial \text{tr}\left({\tilde{\chi }}^{\text{T}}\upsilon \gamma^{\text{T}}\right)+\partial \text{tr}\left(\tilde{\chi }\gamma \gamma^{\text{T}}{\tilde{\chi }}^{\text{T}}\right)\right)/\partial \tilde{\chi }$. It can be derived from the function derivation rule and Lemma 2 that

(20) $$\begin{array}{c} \frac{\partial F_{\text{RTD}}}{\partial \tilde{\chi }}=\varepsilon \left(\tilde{\chi }\gamma \gamma^{\text{T}}-\upsilon \gamma^{\text{T}}\right), \end{array}$$

where *ε* is a constant, *ε*=2. Let $\left(\partial F_{\text{RTD}}/\partial \tilde{\chi }\right)$ be zero. We can get the final solution of Problem 3 by combining formula (12).

(21) $$\begin{array}{c} \tilde{\chi }=\left(\chi \overset{K}{\underset{k}{\prod }}\times_{k}C_{k}\right)\overset{K}{\underset{k}{\prod }}\times_{k}D_{k}^{\text{T}}. \end{array}$$

Algorithm 2 implements RTD by using Algorithm 1. Function TSPA in line 1 represents the implementation of Algorithm 1, and the inputs are *χ*, *η*, and *w*_*η*_. Function F_RTD in line 2 shows the calculation of $\tilde{\chi }$ using equations (18)–(21). Finally, the core tensor of *χ* is obtained by using Algorithm 2, which approximates the restricted tensor η on the layer of tensor structure and elements information.

### 4.2. rdRNN

This study proposes a new RNN called rdRNN. Unlike traditional RNN, rdRNN sets up a new gate based on the bidirectional RNN. The new gate uses the similarity matrix between samples as a parameter of the deep neural network training model. Compared with the original bidirectional RNN, the classification result of rdRNN is more accurate and stable. For the intelligent judgment of legal cases, the original deep neural network method does not consider the correlation between legal cases. This disregard may lead to bias in the final case classification. For example, the verdict of a legal case is inconsistent with the description of the case. To solve this problem, rdRNN fully considers the judgment results of legal cases that are similar to the case to be judged. rdRNN uses these results as a parameter of the deep neural network training model and realizes an efficient and accurate classification of multiple accusations in legal cases.

The following section shows the training of rdRNN's deep neural network while using Rmsprop as its optimization function:

- We use the dataset $\left\{{\tilde{\chi }}^{\left(n\right)},L^{\left(n\right)}\right\}$ and the restricted tensor *η* as inputs of rdRNN. ${\tilde{\chi }}^{\left(n\right)}$ is the core tensor of *χ*^(*n*)^, which represents the legal case. ${\tilde{\chi }}^{\left(n\right)}$ is obtained by the RTD algorithm with *χ*^(*n*)^ and *η* as its inputs. *L*^(*n*)^ represents the category label of *χ*^(*n*)^ according to the judgment result of legal case.
- In this study, we combine rdRNN with the softmax layer to complete the classification of legal cases. For sample *χ*^(*n*)^, assuming *h*^(*n*)^ is the output vector of rdRNN, the softmax layer implements the mapping of *h*^(*n*)^ to the legal case category *L*^(*n*)^.
- We use cross entropy croen_F as the loss function to update rdRNN. rdRNN uses its forward propagation algorithm and error backpropagation formulas to iterate over the values of parameters in neural networks, such as weight matrices {*w*_*d*_} and bias terms {*b*_*d*_} that are associated with relationship gate, and restricted tensor *η*, where *d* is the number of hidden layers.
- We select Adam as the optimization function of rdRNN, and Rmsprop completes the optimization and calculation of parameters {*w*_*d*_}, {*b*_*d*_}, and *η* by using ∂crosen_F/∂*w*  _*d*_, ∂crosen_F/∂*b*  _*d*_, and ∂crosen_F/∂*η*.

#### 4.2.1. Calculation of Forward Propagation in rdRNN

In this study, we fully consider the relationship between legal cases and set up a new gate to complete the classification of legal cases, eliminate contingency errors as much as possible, and avoid inconsistencies between the predicted judgment result and the actual case. Relationship control gate *r*_*t*_ is used to control the similar relationship between legal cases. *r*_*t*_ helps the rdRNN deep neural network make an intelligent judgment by using the judgment results of cases that are similar to the case to be judged.

rdRNN can be divided into forward and backward LSTM. These networks do not have obvious differences, except for the opposite propagation direction. In the case of rdRNN forward LSTM propagation network, the formal description of relationship control gate *r*_*t*_ is as follows:

(22) $$\begin{array}{c} r_{t}=\sigma \left(w_{r}\left[\begin{array}{c} h_{t-1}\\ x_{t} \end{array}\right]+b_{r}\right), \end{array}$$

where *w*_*r*_ and *b*_*r*_ are the weight matrix and bias term of the relational control gate *r*_*t*_, respectively, *σ* is the activation function, i.e., the sigmoid function, *h*_*t*−1_ is the output unit state of the neuron at time *t* − 1, and *x*_*t*_ is the input value of the neuron at time *t*.

In the forward LSTM network, the output of each neuron at time *t* is calculated by the following formula:

(23) $$\begin{array}{c} \left[\begin{array}{c} r_{t}\\ f_{t}\\ i_{t}\\ o_{t}\\ {\tilde{c}}_{t} \end{array}\right]=\left[\begin{array}{c} \sigma \left({\text{net}}_{r,t}\right)\\ \sigma \left({\text{net}}_{f,t}\right)\\ \sigma \left({\text{net}}_{i,t}\right)\\ \sigma \left({\text{net}}_{o,t}\right)\\ \tanh\left({\text{net}}_{\tilde{c},t}\right) \end{array}\right]=\left[\begin{array}{c} \sigma \\ \sigma \\ \sigma \\ \sigma \\ \tanh \end{array}\right]\left(\left[\begin{array}{c} w_{rh},w_{rx}\\ w_{fh},w_{fx}\\ w_{ih},w_{ix}\\ w_{oh},w_{ox}\\ w_{ch},w_{cx} \end{array}\right]\cdot \left[\begin{array}{c} h_{t-1}\\ x_{t} \end{array}\right]+\left[\begin{array}{c} b_{r}\\ b_{f}\\ b_{i}\\ b_{o}\\ b_{c} \end{array}\right]\right), \end{array}$$

where *r*_*t*_, *f*_*t*_, *i*_*t*_, and *o*_*t*_ are the relational control, forget, input, and output gates, respectively; ${\tilde{c}}_{t}$ is the unit status of current inputs; net_*r*,*t*_, net_*f*,*t*_, net_*i*,*t*_, and net_*o*,*t*_ are the weighted inputs of their corresponding gates at time *t*; net_*c*,*t*_ is the weighted inputs of input state generation function tanh; *σ* is the activation function, i.e., the sigmoid function, *σ*(*x*)=1/(1+*e*^−*x*^); tanh is the hyperbolic tangent function, tanh(*x*)=(*e*^*x*^ − *e*^−*x*^)/(*e*^*x*^+*e*^−*x*^); *w*_*r*_ is the weight matrix of relationship control gate *r*_*t*_, *w*_*r*_=[*w*_*rh*_, *w*_*rx*_]; *w*_*f*_, *w*_*i*_, and *w*_*o*_ are expressed in the same manner as *w*_*r*_; and *b*_*r*_, *b*_*f*_, *b*_*i*_, and *b*_*o*_ are the bias terms of their corresponding activation functions.

Subsequently, the unit state of the current moment *c*_*t*_ is calculated by *f*_*t*_, *c*_*t*−1_, *i*_*t*_, and ${\tilde{c}}_{t}$. The calculation formula is expressed as follows:

(24) $$\begin{array}{c} c_{t}=f_{t}*c_{t-1}+i_{t}*{\tilde{c}}_{t}. \end{array}$$

The final output of the forward LSTM neural network at time *t* is calculated by *o*_*t*_, *c*_*t*_, and *r*_*t*_ and the similar list of *x*. It is described as follows:

(25) $$\begin{array}{c} h_{t}=o_{t}*\;\;\tanh\left(c_{t}\right)+r_{t}*\left(\frac{1}{\left|\text{List}\left(x\right)\right|}\underset{x_{0}\in \text{List}\left(x\right)}{\sum }\text{Sim}\left(x_{0},x\right)h_{x_{0}}\right), \end{array}$$

where List(*x*) is composed of legal cases where the similarity with *x* is greater than a threshold Max_sim so far. *h*_*x*_0__ refers to the output of the forward LSTM neural network that corresponds to legal case *x*_0_. Sim(·) is a function that calculates the similarity between legal cases. In this study, we set function Sim as the weight of the Euclidean distance and the cosine distance between legal cases.

(26) $$\begin{array}{c} \text{Sim}\left(x,x_{0}\right)=D_{\text{Euclidean}}\left(x,x_{0}\right)+w_{d}D_{\text{cosine}}\left(x,x_{0}\right), \end{array}$$

where *D*_Euclidean_ and *D*_cosine_ refer to the Euclidean distance and the cosine distance between the vectors *x* and *x*_0_, respectively. *w*_*d*_ is the weight matrix.

#### 4.2.2. Calculation of Backpropagation in rdRNN

In this section, we describe in detail the backpropagation algorithm of the rdRNN neural network, including the backpropagation of the error along time and the hidden layer. In rdRNN, forward and backward LSTM neural networks have the same principle in the backpropagation algorithm. Therefore, this section mainly uses forward LSTM as an example.

Given the error term at time *tδ*_*t*_, *δ*_*t*_=(∂crosen_F/∂*h*_*t*_). Calculation of the backpropagation algorithm of the error term along time is to calculate the value of *δ*_*t*−1_=(∂crosen_F/∂*h*_*t*−1_). The full derivative formula shows that

(27) $$\begin{array}{c} \delta_{t-1}=\frac{\partial \text{crosen\_F}}{\partial h_{t}}\frac{\partial h_{t}}{\partial h_{t-1}}. \end{array}$$

Equations (23)–(25) show that *r*_*t*_, *f*_*t*_, *i*_*t*_, *o*_*t*_, and ${\tilde{c}}_{t}$ are all functions of *h*_*t*−1_. Then, we can obtain

(28) $$\begin{array}{c} \left\{\begin{array}{c} \delta_{o,t}=\delta_{t}\frac{\partial h_{t}}{\partial o_{t}}\frac{\partial o_{t}}{\partial {\text{net}}_{o,t}},\\ \delta_{f,t}=\delta_{t}\frac{\partial h_{t}}{\partial c_{t}}\frac{\partial c_{t}}{\partial f_{t}}\frac{\partial f_{t}}{\partial {\text{net}}_{f,t}},\\ \delta_{i,t}=\delta_{t}\frac{\partial h_{t}}{\partial c_{t}}\frac{\partial c_{t}}{\partial i_{t}}\frac{\partial i_{t}}{\partial {\text{net}}_{i,t}},\\ \delta_{\tilde{c},t}=\delta_{t}\frac{\partial h_{t}}{\partial c_{t}}\frac{\partial c_{t}}{\partial {\tilde{c}}_{t}}\frac{\partial {\tilde{c}}_{t}}{\partial {\text{net}}_{\tilde{c},t}},\\ \delta_{r,t}=\delta_{t}\frac{\partial h_{t}}{\partial r_{t}}\frac{\partial r_{t}}{\partial {\text{net}}_{r,t}}, \end{array}\right.\\ \left\{\begin{array}{c} \frac{\partial {\text{net}}_{o,t}}{\partial h_{t-1}}=w_{oh},\\ \frac{\partial {\text{net}}_{f,t}}{\partial h_{t-1}}=w_{fh},\\ \frac{\partial {\text{net}}_{i,t}}{\partial h_{t-1}}=w_{ih},\\ \frac{\partial {\text{net}}_{\tilde{c},t}}{\partial h_{t-1}}=w_{ch},\\ \frac{\partial {\text{net}}_{r,t}}{\partial h_{t-1}}=w_{rh}. \end{array}\right. \end{array}$$

The formula on the left represents the variable declaration, and the formula on the right is calculated from equation (23). According to equations (27) and (28), we can further derive the following formula:

(29) $$\begin{array}{c} \delta_{t-1}=\delta_{o,t}w_{oh}+\delta_{f,t}w_{fh}+\delta_{i,t}w_{ih}+\delta_{\tilde{c},t}w_{ch}+\delta_{r,t}w_{rh}, \end{array}$$

where *δ*_*o*,*t*_=*δ*_*t*_*∗*  tanh(*c*_*t*_)*∗o*_*t*_*∗*(1 − *o*_*t*_), *δ*_*f*,*t*_=*δ*_*t*_*∗o*_*t*_*∗*(1 − tanh (*c*_*t*_)^2^)*∗c*_*t*−1_*∗f*_*t*_*∗*(1 − *f*_*t*_), $\delta_{i,t}=\delta_{t}*o_{t}*\left(1-\tanh{\left(c_{t}\right)}^{2}\right)*{\tilde{c}}_{t}*i_{t}*\left(1-i_{t}\right)$, $\delta_{\tilde{c},t}=\delta_{t}*o_{t}*\left(1-\tanh\;{\left(c_{t}\right)}^{2}\right)*i_{t}*\left(1-{\tilde{c}}_{t}^{2}\right)$. According to relationship control gate *r*_*t*_, equations (23) and (25), we determine that

(30) $$\begin{array}{c} \delta_{r,t}=\delta_{t}\;*\;\left(\frac{1}{\left|\text{List}\left(x\right)\right|}\underset{x_{0}\in \text{List}\left(x\right)}{\sum }\text{Sim}\left(x_{0},x\right)h_{x_{0}}\right)\;*\;r_{t}\;*\;\left(1-r_{t}\right). \end{array}$$

From equation (29), we can finally figure out the calculation method of the error term in rdRNN is passed from the current moment *t* to any time *k*.

Then, we describe in detail the transmission of error between the hidden layers of rdRNN. The error term of the *l*th hidden layer in rdRNN is assumed to be the partial derivative of the error function crosen_F versus the weighted input net^*l*^. In rdRNN, the input of the *l*th hidden layer at time *t* is *x*_*t*_^*l*^.

(31) $$\begin{array}{c} x_{t}^{l}=\text{active}F^{l-1}\left({\text{net}}_{t}^{l-1}\right), \end{array}$$

where active*F*^*l*−1^ denotes the activation function of the *l* − 1th hidden layer in rdRNN and net_*t*_^*l*−1^ denotes the weighted input of the *l* − 1th hidden layer at time *t*.Given the error term of the *l*th hidden layer at time *tδ*_*t*_^*l*^, *δ*_*t*_^*l*^=(∂crosen_F/∂net_*t*_^*l*^), the calculation of error propagation between hidden layers is to figure out the value of *δ*_*t*_^*l*−1^, where

(32) $$\begin{array}{c} \delta_{t}^{l-1}=\frac{\partial \text{crosen\_F}}{\partial {\text{net}}_{t}^{l-1}}. \end{array}$$

According to equations (23), (31), and (32), net_*r*,*t*_^*l*^, net_*f*,*t*_^*l*^, net_*i*,*t*_^*l*^, net_*o*,*t*_^*l*^, and ${\text{net}}_{\tilde{c},t}^{l}$ are all functions of *x*_*t*_^*l*^, and *x*_*t*_^*l*^ is a function of net_*t*_^*l*−1^. Therefore, the full derivative formula shows that

(33) $$\begin{array}{c} \delta_{t}^{l-1}=\frac{\partial \text{crosen\_F}}{\partial {\text{net}}_{r,t}^{l}}\frac{\partial {\text{net}}_{r,t}^{l}}{\partial x_{t}^{l}}\frac{\partial x_{t}^{l}}{\partial {\text{net}}_{t}^{l-1}}+\frac{\partial \text{crosen\_F}}{\partial {\text{net}}_{f,t}^{l}}\frac{\partial {\text{net}}_{f,t}^{l}}{\partial x_{t}^{l}}\frac{\partial x_{t}^{l}}{\partial {\text{net}}_{t}^{l-1}}+\frac{\partial \text{crosen\_F}}{\partial {\text{net}}_{i,t}^{l}}\frac{\partial {\text{net}}_{i,t}^{l}}{\partial x_{t}^{l}}\frac{\partial x_{t}^{l}}{\partial {\text{net}}_{t}^{l-1}}+\frac{\partial \text{crosen\_F}}{\partial {\text{net}}_{o,t}^{l}}\frac{\partial {\text{net}}_{o,t}^{l}}{\partial x_{t}^{l}}\frac{\partial x_{t}^{l}}{\partial {\text{net}}_{t}^{l-1}}+\frac{\partial \text{crosen\_F}}{\partial {\text{net}}_{\tilde{c},t}^{l}}\frac{\partial {\text{net}}_{\tilde{c},t}^{l}}{\partial x_{t}^{l}}\frac{\partial x_{t}^{l}}{\partial {\text{net}}_{t}^{l-1}}. \end{array}$$

The following formula can be obtained by further calculation:

(34) $$\begin{array}{c} \delta_{t}^{l-1}=\left(\delta_{r,t}^{l}w_{rx}+\delta_{f,t}^{l}w_{fx}+\delta_{i,t}^{l}w_{ix}+\delta_{o,t}^{l}w_{ox}+\delta_{\tilde{c},t}^{l}w_{cx}\right)*\text{gra}\left(\text{active}F^{l-1}\left({\text{net}}_{t}^{l-1}\right)\right), \end{array}$$

where gra(active*F*^*l*−1^(net_*t*_^*l*−1^)) is the derivative of function active*F*^*l*−1^ at net_*t*_^*l*−1^.

According to equations (27)–(34), we can derive the partial derivative of the loss function crosen_F to the weight matrix set {*w*  _*d*_} and the bias term set {*b*  _*d*_} in rdRNN. Given that ∂crosen_F/∂*w*_*rh*_=∑_*j*=1_^*t*^∂crosen_F/∂*w*_*rh*,*j*_=∑_*j*=1_^*t*^(∂crosen_F/∂net_*r*,*j*_)(∂net_*r*,*j*_/∂*w*_*rh*,*j*_), we obtain

(35) $$\begin{array}{c} \left\{\begin{array}{c} \frac{\partial \text{crosen\_F}}{\partial w_{rx}}=\overset{t}{\underset{j=1}{\sum }}\delta_{r,j}h_{j-1},\\ \frac{\partial \text{crosen\_F}}{\partial w_{fx}}=\overset{t}{\underset{j=1}{\sum }}\delta_{f,j}h_{j-1},\\ \frac{\partial \text{crosen\_F}}{\partial w_{ix}}=\overset{t}{\underset{j=1}{\sum }}\delta_{i,j}h_{j-1},\\ \frac{\partial \text{crosen\_F}}{\partial w_{ox}}=\overset{t}{\underset{j=1}{\sum }}\delta_{o,j}h_{j-1},\\ \frac{\partial \text{crosen\_F}}{\partial w_{cx}}=\overset{t}{\underset{j=1}{\sum }}\delta_{\tilde{c},j}h_{j-1}, \end{array}\right.\\ \left\{\begin{array}{c} \frac{\partial \text{crosen\_F}}{\partial b_{r}}=\overset{t}{\underset{j=1}{\sum }}\delta_{r,j},\\ \frac{\partial \text{crosen\_F}}{\partial b_{f}}=\overset{t}{\underset{j=1}{\sum }}\delta_{f,j},\\ \frac{\partial \text{crosen\_F}}{\partial b_{i}}=\overset{t}{\underset{j=1}{\sum }}\delta_{i,j},\\ \frac{\partial \text{crosen\_F}}{\partial b_{o}}=\overset{t}{\underset{j=1}{\sum }}\delta_{o,j},\\ \frac{\partial \text{crosen\_F}}{\partial b_{c}}=\overset{t}{\underset{j=1}{\sum }}\delta_{\tilde{c},j}, \end{array}\right.\\ \left\{\begin{array}{c} \frac{\partial \text{crosen\_F}}{\partial w_{rx}}=\delta_{r,j}x_{t},\\ \frac{\partial \text{crosen\_F}}{\partial w_{fx}}=\delta_{f,j}x_{t},\\ \frac{\partial \text{crosen\_F}}{\partial w_{ix}}=\delta_{i,j}x_{t},\\ \frac{\partial \text{crosen\_F}}{\partial w_{ox}}=\delta_{o,j}x_{t},\\ \frac{\partial \text{crosen\_F}}{\partial w_{cx}}=\delta_{\tilde{c},j}x_{t}. \end{array}\right. \end{array}$$

#### 4.2.3. Calculation of the Partial Derivative of Loss Function crosen_F to Restricted Tensor *η*

This study proposes a new intelligent method for judging legal cases called RnRTD, which combines rdRNN and RTD to complete the classification of legal cases. In the process of training the RnRTD neural network, a new problem is involved: updating of the value of the restricted tensor *η* so that it can continuously approximate the tensor value that is most beneficial for improving the classification accuracy of the RnRTD algorithm.

The crux to solving this problem is to calculate the partial derivative of the loss function crosen_F to the restricted tensor *η*, that is, ∂crosen_F/∂*η*. Directly solving the value of ∂crosen_F/∂*η* is difficult. We can use the full derivative rule to obtain that

(36) $$\begin{array}{c} \frac{\partial \text{crosen\_F}}{\partial \eta }=\overset{N}{\underset{n}{\sum }}\frac{\partial \text{crosen\_F}}{\partial {\tilde{\chi }}^{\left(n\right)}}\frac{\partial {\tilde{\chi }}^{\left(n\right)}}{\partial \eta }. \end{array}$$

The backward propagation formula of rdRNN shows that $\partial \text{crosen\_F}/\partial {\tilde{\chi }}^{\left(n\right)}=\left(\partial \text{crosen\_F}/\partial {\text{net}}_{1}\right)\left(\partial {\text{net}}_{1}/\partial {\tilde{\chi }}^{\left(n\right)}\right)=\delta_{1}w_{0}$. According to equations (19)–(21), we determine that $\partial {\tilde{\chi }}^{\left(n\right)}/\partial \eta =\partial \left[\left(\chi^{\left(n\right)}\prod_{k}^{K}\times_{k}C_{k}^{\left(n\right)}\right)\prod_{k_{1}}^{K}\times_{k_{1}}{U_{k_{1}}^{\left(n\right)}}^{\text{T}}\right]/\partial \eta$. From equations (14)–(17), we know that *C*_*k*_^(*n*)^ and *D*_*k*_^(*n*)^ are all functions of *η*. Therefore, the function full derivative rule shows that $\partial {\tilde{\chi }}^{\left(n\right)}/\partial \eta =\sum_{k}^{K}\left(\partial {\tilde{\chi }}^{\left(n\right)}/\partial C_{k}^{\left(n\right)}\right)\left(\partial C_{k}^{\left(n\right)}/\partial \eta \right)+\sum_{k}^{K}\left(\partial {\tilde{\chi }}^{\left(n\right)}/\partial D_{k}^{{\left(n\right)}^{\text{T}}}\right)\left({\partial D_{k}^{\left(n\right)}}^{\text{T}}/\partial \eta \right)$, that is

(37) $$\begin{array}{c} \frac{\partial {\tilde{\chi }}^{\left(n\right)}}{\partial \eta }=\overset{K}{\underset{k}{\sum }}\text{cal}C\left(C_{k}^{\left(n\right)}\right)+\overset{K}{\underset{k}{\sum }}\text{cal}D\left({D_{k}^{\left(n\right)}}^{\text{T}}\right), \end{array}$$

while

(38) $$\begin{array}{c} \left\{\begin{array}{c} \text{cal}C\left(C_{k}^{\left(n\right)}\right)=\chi^{\left(n\right)}\overset{K}{\underset{k_{1}\neq k}{\prod }}\times_{k_{1}}C_{k_{1}}^{\left(n\right)}\times_{k}\frac{\partial C_{k}^{\left(n\right)}}{\partial \eta }\overset{K}{\underset{k_{2}}{\prod }}\times_{k_{2}}{D_{K_{2}}^{\left(n\right)}}^{\text{T}},\\ \text{cal}D\left({D_{k}^{\left(n\right)}}^{\text{T}}\right)=\chi^{\left(n\right)}\overset{K}{\underset{k_{1}}{\prod }}\times_{k_{1}}C_{k_{1}}^{\left(n\right)}\times_{k}\frac{{\partial D_{k}^{\left(n\right)}}^{\text{T}}}{\partial \eta }\overset{K}{\underset{k_{2}\neq k}{\prod }}\times_{k_{2}}{D_{K_{2}}^{\left(n\right)}}^{\text{T}}. \end{array}\right. \end{array}$$

Algorithm 3 provides the optimization process of RnRTD proposed in this study. max_iter in line 2 represents the total number of training sessions of RnRTD. batch_size in line 6 represents the number of samples per batch while training RnRTD. The RTD tensor decomposition method in line 7 corresponds to Algorithm 2. rdRNN_forwardprop in line 8 and rdRNN_backprop in line 9 represent forward propagation and error backpropagation algorithms for rdRNN, respectively, which are the implementations of Sections 4.2.1–4.2.3. In line 11, we use the Adam algorithm to realize parameter optimization of RnRTD neural network.

#### 4.2.4. Loss Function crosen_F and Softmax Layer

In Algorithm 3, we use the softmax function softmax to calculate the probability that *χ*^(*n*)^ belongs to each type of legal case according to judgment results.

**Definition 9 .**

Given a set of samples of legal cases and their corresponding outputs of RnRTD {(*χ*^(*n*)^, *S*^(*n*)^)}, the probability that *χ*^(*n*)^ belongs to each type of legal case is calculated by

(39) $$\begin{array}{c} L_{1q}^{\left(n\right)}=\text{softmax}\left(S^{\left(n\right)}\right)=\frac{e^{S_{q}^{\left(n\right)}}}{\sum_{r}^{Q}e^{S_{r}^{\left(n\right)}}}, \end{array}$$

where *L*_1*q*_^(*n*)^ represents the *q*th element of *L*_1_^(*n*)^.

In this study, cross entropy is used as the loss function crosen_F to calculate the error of RnRTD. We define crosen_F as follows:

**Definition 10 .**

A set of samples of legal cases and their corresponding legal case types {(*χ*^(*n*)^, *L*^(*n*)^)} is given. The predicted legal case category of *χ*^(*n*)^ is *L*_1_^(*n*)^, which is calculated by RnRTD, and then

(40) $$\begin{array}{c} \text{crosen\_F}\left(\left\{L^{\left(n\right)}\right\},\left\{L_{1}^{\left(n\right)}\right\}\right)=\overset{N}{\underset{n}{\sum }}\overset{Q}{\underset{q}{\sum }}L_{q}^{\left(n\right)}\text{log}L_{1q}^{\left(n\right)}, \end{array}$$

where *N* represents the number of samples of legal cases and *q* represents the dimension of *L*^(*n*)^ and *L*_1_^(*n*)^, that is, the number of types of legal cases.

## 5. Results

### 5.1. Description of Experimental Data

We use nearly 1.8 million historical legal cases obtained from a Chinese refereeing study network. These legal cases involve more than 200 types of accusations, including theft, intentional assault, smuggling, fraud, and deliberate destruction of public property. Approximately 400,000 cases involve theft, and about 200,000 cases involve intentional assault. The number of accusations involved in each case ranges from 1 to 23.

**Figure 8:**
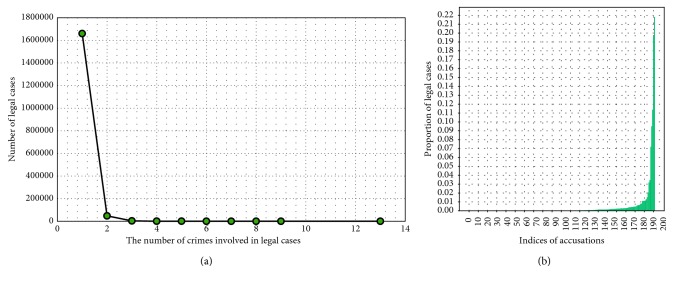
Statistics on allegations in legal cases.

Figure 8(a) shows the distribution of various accusations in the legal case data used in this study. The abscissa indicates the accusation index. For example, index 1 corresponds to bribery, and index 2 corresponds to rape. The ordinate indicates the proportion of cases involving the accusation that occupy the overall cases. Figure 8(a) shows that the number of cases involving theft is the highest in the database used in this article.

Figure 8(b) shows the distribution of the number of accusations involved in each case. The abscissa indicates the number of accusations involved in the case, and the ordinate indicates the proportion of cases in the corresponding number of accusations. Figure 8(b) shows that the highest number of accusations involves three cases.

### 5.2. Baseline Approaches

Given that multiaccusation judgment based on deep neural network and tensor decomposition is rarely studied, according to the limited tensor decomposition method RTD and the relation-based recurrent neural network rdRNN, we use the following method for a comparison with RnRTD proposed in this study:

- This study uses a series of deep neural networks based on convolutional or cyclic neural networks, including TextCNN, TextCNN attention, TextRNN, TextRNN attention, LSTM, and Bi-LSTM, as comparison methods for RnRTD.
- This study uses deep neural networks with only the RTD tensor decomposition layer as comparison methods for RnRTD. Through experiments, we can derive the contribution of the RTD tensor decomposition layer to all deep neural networks.
- This study uses neural networks with only the rdRNN method as comparison methods for RnRTD. Through these comparison experiments, we can derive the contribution of the relationship-based rdRNN strategy to all RNNs.

### 5.3. Data Preprocessing

In this study, the data preprocessing operation can be divided into two parts, namely, the modular representation of legal cases and the construction of the original tensor. Our legal case data preprocessing process can be described as follows:

- We organize each case in the legal case database into our preestablished case model, which divides the original case file into the defendant's statement, the plaintiff's statement, the content of the public prosecutions allegations, and the court's judgment.
- We filter and clean the contents of each module in the legal cases, extract the words that are meaningful to our multiaccusation judgment method, and filter out redundant words, stop words, noisy words, and modal particles.
- We train the word-to-vector model to obtain the word vector of the aforementioned vocabulary. Then, we obtain a matrix representation of each case module and derive the tensor representation of the entire legal case.

For Step (1), each case module may be spread across different paragraphs, and cases in different regions have different case descriptions. We extract and integrate them separately to arrive at a modular representation of the cases based on the description rules of case documents in each region. For Step (2), the extraction and filtering of the vocabulary in legal cases often requires professional legal background knowledge; otherwise, error filtering will occur. We filter words in legal case modules by using the legal professional vocabulary and the stop word list. For Step (3), word vectors are the basis for the accuracy of the entire deep neural network method. We use a number of Chinese corpus, such as corpus on Baidu Encyclopedia, Zhihu Questions and Answers, Sohu News, and Sina Weibo, to train the word-to-vector model.

The tensor representation of legal cases and the subsequent deep neural network classification method require each case to have the same number of words, and the number of words in 95% of the cases is below 300. Therefore, we perform a padding operation for cases where the number of words is less than 300. For cases with a vocabulary number greater than 300, we use the TF-IDF weight of the vocabulary to tailor the case vocabulary.

### 5.4. Experimental Hyperparameter Setting

This section describes the hyperparameter settings involved in our proposed method. These settings include the restricted tensor *η* and weight matrix *W*_*η*_ in RTD and the size of the similar case list in the rdRNN method (i.e., the size of list |*∗*| in equation (25)).

The setting of restricted tensor *η* directly affects the convergence speed and accuracy of RnRTD. Our experiments show that a large rank of restricted tensor *η* corresponds to a high accuracy of the subsequent deep neural network algorithms. Conversely, a strong linear relationship between column or row vectors in *η* results in a low accuracy of the subsequent classification algorithms.

In this study, weight matrix *W*_*η*_ is used to scale the elements of the last mode in the original tensors. *W*_*η*_ adjusts the weights of certain words in the legal cases. For different accusations, the same vocabulary may have different weights in different types of cases. For example, derailment occupies a large and small weight in cases that involve bigamy and smuggling, respectively.

The size of the similar case list in rdRNN is an important indicator that determines the impact of the relationship between cases on the final classification result. If the length of the similar case list is set too long, then it is equivalent to strengthening the weak similarity between cases and weakening the strong similarity between cases. Furthermore, if the length of the similar case list is set too short, then it is equivalent to weakening the weak similarity between cases and strengthening the strong similarity between cases. After many experiments, we set the case similar list length to 50.

### 5.5. Experimental Results and Analysis

This section shows the superiority of the proposed RnRTD method for multiple accusations in legal cases relative to the baseline listed in Section 5.2 and provides the corresponding analysis.

**Figure 9:**
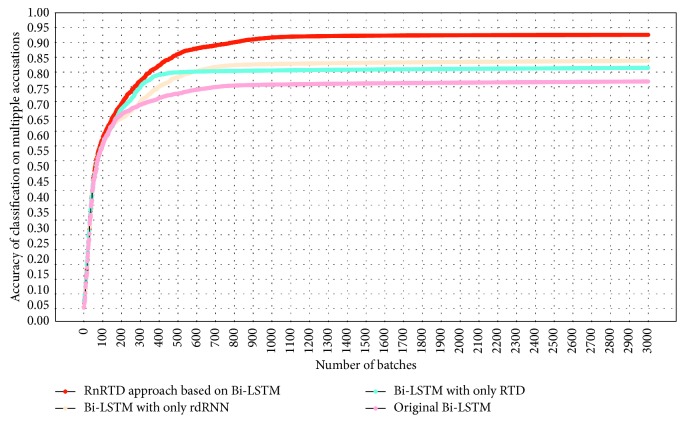
Experimental results of RnRTD based on Bi-LSTM.

Figure 9 shows a series of experimental results based on Bi-LSTM. The abscissa indicates the number of batch iterations, and the ordinate indicates the accuracy of the multiaccusation judgment methods in legal cases. In contrast with the original Bi-LSTM method and Bi-LSTM with only the rdRNN layer, Bi-LSTM with only RTD achieves stable accuracy at the highest speed as the number of batches increases.

The characteristics of RTD are important factors in the aforementioned phenomenon. On the basis of the restricted tensor η, RTD extracts the tensor elements and structure information that are most relevant to the multiaccusation judgment of legal cases from the original tensor. The weight of the vocabulary unrelated to a particular accusation is considerably weakened, and the weight of the vocabulary associated with a particular accusation is strengthened. The tensor dimension is greatly reduced, and the influence of irrelevant vocabulary on the classification algorithm is reduced. Subsequent neural network algorithms continuously iterate and optimize the restricted tensor and continuously adjust and correct the element values of the core tensor. RTD optimizes the original deep neural network algorithm from the lexical level.

In Figure 9, as the number of batches increases, the accuracy of Bi-LSTM with only the rdRNN layer becomes ultimately higher than that of Bi-LSTM with only the RTD layer. The reason is that rdRNN fully considers the similarity between different cases and has better discrimination for similar cases expressed by different language description methods. By setting the appropriate similar case list size, rdRNN fully considers cases that are similar to the current case and weighs their corresponding output states according to their similarity. rdRNN corrects and optimizes the original deep neural network from the case level.

**Figure 10:**
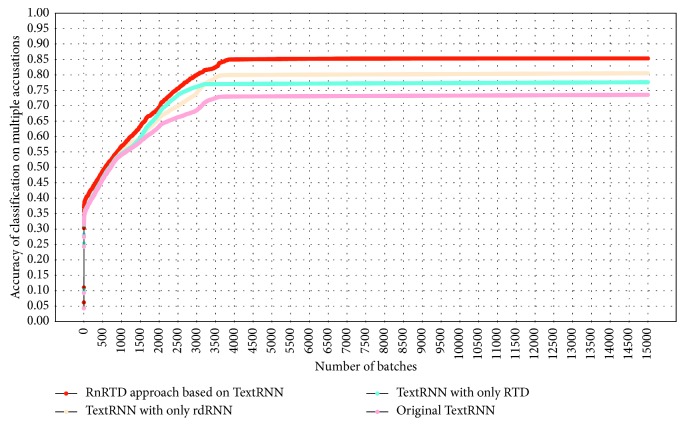
Experimental results of RnRTD based on TextRNN.

Figure 10 shows the experimental results of the TextRNN-based RnRTD method. Similar to what is shown in Figure 9, TextRNN with only the RTD layer has the highest convergence speed as the number of batches increases compared with the original TextRNN and TextRNN with only the rdRNN layer.

The accuracy of the deep neural network method with only the rdRNN layer is not always higher than that with only the RTD layer. Although the rdRNN layer implements the correction and optimization of subsequent classification algorithms at the case level through the setting of similar case lists, the RTD layer also optimizes classification algorithms at the vocabulary level by setting the restricted tensor. Both methods achieve the final accuracy optimization but have different effects for various contexts. RnRTD combines the advantages of RTD and rdRNN to achieve rapid convergence and high classification accuracy.

Table 2 provides an experimental comparison of RnRTD methods based on multiple deep neural networks. RnRTD remarkably improves the classification accuracy of original neural networks for the classification of multiple accusations in legal cases. RTD and rdRNN layers also have considerable optimization effects on the original neural networks. RTD is applicable to all deep neural networks and can extract the main information carried by the data at the input layer to realize dimension reduction. rdRNN is an optimization strategy that is suitable for RNNs. It fully considers the similarity between cases within a certain period and optimizes algorithms at the case level. For algorithms based on convolutional neural networks, we remove the relational control gate in rdRNN while retaining the similar case list. Then, the optimization of these algorithms is realized by the rdRNN layer.

The convolutional neural network is less effective than RNN because legal case data are time series data. In addition, the attention layer only changes the encoding of the input and does not change the structure of neural networks. For the problem of judgment for multiple accusations in legal cases, the attention layer is still difficult to compensate for due to the lack of timing information of TextCNN and the gradient disappearance and gradient explosion of the TextRNN algorithm. From the perspective of the rdRNN layer, GRU has fewer adjustable parameters than LSTM and Bi-LSTM, and optimization on the restricted tensor is relatively limited. Therefore, the Bi-LSTM neural network with RnRTD performs better than other neural network algorithms.

## 6. Discussion

In this study, we propose a new method for multiaccusation judgment in legal cases called RnRTD. RnRTD is a multilabel classification method based on tensor decomposition and RNNs. RnRTD consists of the tensor decomposition method with constraints and relation-driven RNN.

We propose a tensor decomposition method with constraints, namely, RTD. We use this method to extract the tensor structure and element information that are most favorable to the subsequent classification algorithm from the original tensor that represents the legal case. RTD continuously corrects and optimizes the values of elements in the core tensor through the weight matrix and restricted tensor; hence, it continues to improve the classification accuracy of the neural network. RTD optimizes neural network classification algorithms at the lexical level. We also propose a relation-driven RNN strategy called rdRNN. Unlike traditional recurrent and LSTM neural networks, rdRNN sets up a new gating switch, that is, the similarity list window. It controls the impact of cases similar to the current case on the output status of the current case. rdRNN optimizes neural network classification algorithms at the case level.

According to our experimental results, the RTD layer and the relation-driven cyclic neural network rdRNN have remarkable optimization effects on various deep neural network algorithms. However, no obvious relationship exists between the two. RTD and rdRNN have their own advantages in different contexts. In Figures 9 and 10, the accuracy of rdRNN is higher than that of RTD. The accuracy of rdRNN is not always higher than that of RTD. RTD achieves stable accuracy the fastest as the number of batches increases in both figures. The reason is the principal component extraction and dimensionality reduction of RTD itself.

RTD is suitable for almost all deep neural networks. It performs principal component extraction and dimensionality reduction on the original data at the input layer. It is similar to traditional principal component analysis methods, such as PCA [20] and SVD [21]. Several decomposition methods [22], such as Tucker and CP [23], are currently used for high-dimensional data. These methods extract the main elements and structural information of the matrix or tensor at the logical level according to the linear relationship of the elements in the matrix or tensor. However, the resulting new matrix or tensor structure is often unexplained. According to the traditional matrix or tensor decomposition method, supervising the completion of the principal component extraction work is difficult.

The proposed restricted tensor provides interpretability for the tensor decomposition operation. Under the influence of the restricted condition tensor, RTD retains the information in the original tensor that is beneficial to the subsequent neural network and removes useless information. For the overall classification algorithm, RTD reduces the weight of weak correlation information and improves the influence of strong correlation information on the classification model. In addition, the subsequent deep neural network algorithm will continuously update and optimize the constraints in RTD to guide the core tensor to retain information that is conducive for the classification model. RTD optimizes the classification model at the vocabulary level by combining the weight matrix with the restricted tensor.

rdRNN fully considers the similarities between cases and uses it as a factor that influences the output status of the current case. rdRNN optimizes the entire classification model at the case level. Generally, different regions may use different legal case description vocabularies, and rdRNN sets the output status of similar historical cases within a certain period as the reference value of the current case output state by setting a similar case window. Moreover, it sets the weight according to the similarity. RnRTD combines RTD and rdRNN to optimize the classification results from the perspective of case and vocabulary. When we use rdRNN to optimize algorithms based on the convolutional neural network, we remove the relationship control gate, retain only the similar case list in rdRNN, and realize the optimization operation of the rdRNN layer on the neural network.

## 7. Conclusion

In this study, we propose a new method for judging multiple crimes in legal cases, namely RnRTD. RnRTD consists of RTD and rdRNN. RTD is a tensor decomposition method with constraints. RTD decomposes the original tensor that represents a legal case into a core tensor under the guidance of restricted tensor. The resulting core tensor represents the main tensor structure and element information that is most favorable for improving the accuracy of subsequent classification algorithm. We propose the rdRNN algorithm and train it using obtained core tensors. rdRNN guides the tensor decomposition process in RTD by continuously optimizing the restricted tensor and finally makes RTD develop in the direction that is most beneficial to improve the classification accuracy of rdRNN.

Nevertheless, this study has several problems. For example, even with the RTD tensor decomposition layer, algorithms based on RNNs usually run very slowly. In our future work, we will attempt to reduce the computational complexity of the algorithm and increase its speed.

## Figures and Tables

**Algorithm 1:**
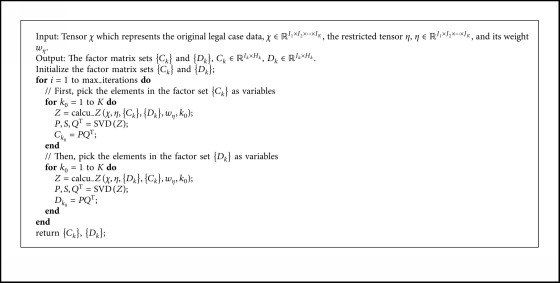
The solution of Problem 2 by using ALS Algorithm.

**Algorithm 2:**
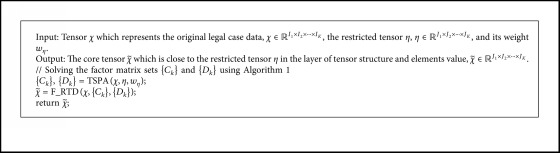
The restricted tensor decomposition method.

**Algorithm 3:**
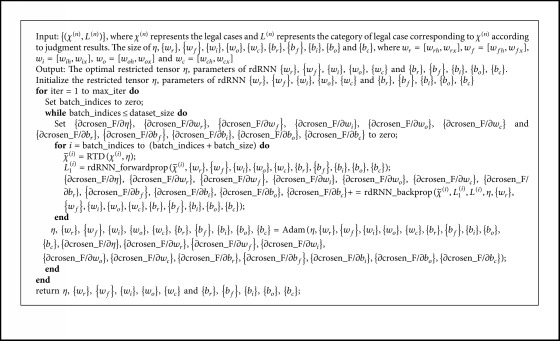
The framework of RnRTD algorithm.

**Table 1:** Symbols.

Symbol	Definition
*χ*, *X*, **x**, *x*	Tensor, matrix, vector, scalar
*A* ^T^	Transpose of matrix *A*
*χ*(*i*_1_,…, *i*_*N*_)	The (*i*_1_,…, *i*_*N*_)th entry of *χ*, same for vectors and matrices
*A*(:,*i*)	The *i*th column of matrix *A*
∘	Outer product
*∗*	Elementwise multiplication
⊗	Kronecker product
⊙	Khatri–Rao product
×^*n*^	The *n*-mode stretch
×_*n*_	The *n*-mode product
*χ*_(*n*)_	The *n*-mode matricization of tensor *χ*
*χ*_F_	The Frobenius norm of tensor *χ*

**Table 2:** Performance of RnRTD methods based on multiple deep neural networks.

Optimization method	Basic algorithm						
	TextCNN	TextRNN	TextCNN attention	TextRNN attention	LSTM	Bi-LSTM	GRU
Original method	0.70	0.73	0.74	0.76	0.75	0.77	**0.79**
Only with RTD	0.73	0.77	0.79	0.82	0.80	0.81	**0.83**
Only with rdRNN	0.74	0.80	0.82	0.83	0.83	**0.84**	0.82
With RnRTD	**0.80**	**0.85**	**0.85**	**0.90**	**0.91**	**0.93**	**0.91**

## Data Availability

The legal cases data after processing used to support the findings of this study are currently under embargo while the research findings are commercialized. Requests for data, 6/12 months after publication of this article, will be considered by the corresponding author.

## References

[1] Hahn U., Chater N. (1998). *Understanding Similarity: A Joint Project for Psychology, Case-Based Reasoning, and Law*.

[2] Aleven V. (2003). Using background knowledge in case-based legal reasoning: a computational model and an intelligent learning environment. *Artificial Intelligence*.

[3] Bench-Capon T., Sartor G. (2003). A model of legal reasoning with cases incorporating theories and values. *Artificial Intelligence*.

[4] Garcez A. S. A., Lamb L. C., Gabbay D. M. (2009). *Neural-Symbolic Cognitive Reasoning*.

[5] Besold T. R., Garcez A. S. A., Bader S. (2017). Neural-symbolic learning and reasoning: a survey and interpretation. https://arxiv.org/abs/1711.03902.

[6] Garcez A. A., Besold T., de Raedt L. Neural-symbolic learning and reasoning: contributions and challenges.

[7] Garcez A. S. A., Gabbay D. M., Lamb L. C. (2014). A neural cognitive model of argumentation with application to legal inference and decision making. *Journal of Applied Logic*.

[8] Venkateshmurthy M. G., Geetha T. V., Subramanian R. K. (1990). Extraction and representation of facts from legal briefs. *Journal of Intelligent and Robotic Systems*.

[9] Breaux T. D., Antn A. I., Spafford E. H. (2009). A distributed requirements management framework for legal compliance and accountability. *Computers and Security*.

[10] Waterman D. A., Paul J., Peterson M. (1986). Expert systems for legal decision making. *Expert Systems*.

[11] Bartolini R., Lenci A., Montemagni S., Pirrelli V., Soria C. *Automatic Classification and Analysis of Provisions in Italian Legal Texts: A Case Study*.

[12] Joshi S., Deshpande P. M., Hampp T. Improving the efficiency of legal e-discovery services using text mining techniques.

[13] Sulea O.-M., Zampieri M., Malmasi S., Vela M., Dinu L. P., van Genabith J. (2017). Exploring the use of text classification in the legal domain. https://arxiv.org/abs/1710.09306.

[14] Bruninghaus S., Ashley K. D. Progress in textual case-based reasoning: predicting the outcome of legal cases from text.

[15] Welling M., Weber M. (2001). Positive tensor factorization. *Pattern Recognition Letters*.

[16] Williams R. J., Zipser D. (1989). *A Learning Algorithm for Continually Running Fully Recurrent Neural Networks*.

[17] Hansen L. K., Salamon P. (2002). Neural network ensembles. *IEEE Transactions on Pattern Analysis and machine intelligence*.

[18] Gers F. A., Schmidhuber J., Cummins F. Learning to forget: continual prediction with lstm.

[19] Graves A., Schmidhuber J. (2005). Framewise phoneme classification with bidirectional lstm and other neural network architectures. *Neural Networks*.

[20] Oja E. (1997). The nonlinear pca learning rule in independent component analysis. *Neurocomputing*.

[21] Vozalis M., Margaritis K. (2007). Using svd and demographic data for the enhancement of generalized collaborative filtering. *Information Sciences*.

[22] Cichocki A., Zdunek R., I Amari S. (2009). Nonnegative matrix and tensor factorization. *IEEE Signal Processing Magazine*.

[23] Kolda T. G., Bader B. W. (2009). Tensor decompositions and applications. *SIAM Review*.

